# Mental distress links with physical activities, sedentary lifestyle, social support, and sleep problems: A Syrian population cross-sectional study

**DOI:** 10.3389/fpsyt.2022.1013623

**Published:** 2023-01-16

**Authors:** Sarya Swed, Hidar Alibrahim, Haidara Bohsas, Abdulqadir J. Nashwan, Mohamed Elsayed, Mohammad B. Almoshantaf, Saeed A. Kadri, Bisher Sawaf, Mhd Kutaiba Albuni, Elias Battikh, Nashaat K. Elkalagi, Safaa M. Ahmed, Eman M. Ahmed, Mohammad Mehedi Hasan, Muhammad Mainuddin Patwary, Sheikh Shoib, Wael Hafez

**Affiliations:** ^1^Faculty of Medicine, Aleppo University, Aleppo, Syria; ^2^Hamad Medical Corporation, Doha, Qatar; ^3^Department of Psychiatry and Psychotherapy III, University of Ulm, Ulm, Germany; ^4^Department of Psychiatry, School of Medicine and Health Sciences, Carl von Ossietzky University Oldenburg, Oldenburg, Germany; ^5^Department of Neurosurgery, Ibn Al-Nafees Hospital, Damascus, Syria; ^6^Faculty of Medicine, Syrian Private University, Damascus, Syria; ^7^Faculty of Medicine, Damascus University, Damascus, Syria; ^8^Internal and Tropical Medicine Department, Faculty of Medicine, Al Arish University, Al Arish, Egypt; ^9^Faculty of Medicine, Shendi University, Shendi, Sudan; ^10^Department of Obstetrics and Gynecology, Nile Valley University, Khartoum, Sudan; ^11^Department of Biochemistry and Molecular Biology, Faculty of Life Science, Mawlana Bhashani Science and Technology University, Tangail, Bangladesh; ^12^Environment and Sustainability Research Initiative, Khulna, Bangladesh; ^13^Environmental Science Discipline, Life Science School, Khulna University, Khulna, Bangladesh; ^14^Department of Psychiatry, Jawahar Lal Nehru Memorial Hospital, Srinagar, Kashmir, India; ^15^Medical Research Division, Department of Internal Medicine, The National Research Centre, Cairo, Egypt

**Keywords:** mental distress, physical activity, sedentary lifestyle, social support, sleep problems, Syria

## Abstract

**Background:**

Mental diseases are very widespread and difficult to treat, affecting around 12% of the global population in 2019. Since social interaction is crucial to human existence and loneliness has been proven to be a significant predictor of depressive symptoms, it stands to reason that social connection problems would also contribute to depression. Physical inactivity seems to weaken and aggravate insulin tolerance alterations, glucose homeostasis, and plasma triglyceride levels, thereby influencing one's mood and happiness. This suggests that physical inactivity may be a significant risk factor for mental illness. This research contributes to our understanding of the mental health situation in Syria by exploring associations between a set of measurable characteristics that may be adjusted.

**Methods:**

An online quantitative cross-sectional study was conducted between March and April 2022 in Syria, using a structured questionnaire that assesses data on behaviors of health, health in general, wellbeing, and adult population quality of life.

**Results:**

Among 1,224 respondents (371 men and 853 women), women have shown higher levels of mental distress, sleep issues, low engagement in structured activities, and a difficult work environment than men. Women experiencing mental anguish have reported being more sedentary, participating in less scheduled activities, and receiving less social support.

**Conclusions:**

There are observable connections between high sedentary time and women experiencing mental distress. The mental health of Syrian women in distress was associated with a lack of participation in both organized activities and physical exercise in their free time. Furthermore, sleep issues and financial troubles were seen in persons with mental diseases of both males and females.

## Introduction

As per the World Health Organization, mental health is a state of wellbeing in which an individual acknowledges their strengths, can cope with daily problems, is productive and successful, and participates in their community (1). Mental disorders are pretty common and onerous. In 2019, projections predicted that about 12% of the global population would be affected by mental illness (2). Mental disorders have a significant economic burden on society. For example, the cost of having any mental disorder is 9.2 times that of not having one. Therefore, an effective treatment and prevention program is urgently needed to reduce the economic burden (3). Many modifiable risk factors are related to mental disorders, including alcohol, smoking, substance usage, unfavorable assessments of stressful life events, occupational causes, depressive symptoms, Body Mass Index, and hypertension. On the other hand, physical activity, exercising, social support, and the ability to cope were all considered protective factors against mental disorders (4). Even though there is a lot of evidence showing regular physical exercise is associated to decreased mortality rates and various advantages for mental health, only a small percentage of the population actually participates in regular physical activity (5). More need to be given to people's mental health, particularly in relation to their level of physical exercise and other beneficial habits (6). Several studies suggest that organized activities such as singing, dancing, cultural engagement, and various recreational activities can cause emotional feelings, lead to more positivity and effectiveness, and have an essential role in preventing mental illness, reducing depression and anxiety (7, 8). These activities are considered one of the modifiable factors and cornerstone of public health support, including mental health; still, it is insufficient to treat any mental disorder (9). A greater quality of life, more self-confidence, and improved ability to handle anxiety are just some of the psychological benefits that have been associated to participation in physically active activities like sports. It is also possible that it will aid with social development, social involvement, and social integration (10). People who join sports groups or participate in organized leisure time have excellent mental health, and are more attentive and more robust to contemporary life's demands (11). Participating in organized leisure activities allows participants to form social networks where several studies have shown links between social contact and mental health (12). Medical literature has indicated that solitude is a strong predictor of depression symptoms; also, social connectivity is essential to human life, so social relationship impairments are likely to cause depression (13, 14). Poor social communication with people leads to more unhealthy behaviors such as drinking alcohol, smoking, and sleep disorders which put the patient in a vicious cycle (15). Many persons suffering from severe mental illness are characterized by social isolation and a lack of wanted interactions.

Their isolation comes from their inability to establish and maintain relationships, a lack of networking activities, and the stigma related to mental illness (16). Friends are the key to improving mental health and reducing its problems. They play an essential role in emotional support, bearing challenges and pressures, enhancing a sense of value, and creating a feeling of safety (16). In Syria, there is a need for mental health care in combat zones, refugee camps, and among internally displaced people, where the conflict and migration have resulted in a high frequency of trauma and major mental illnesses among Syrians (17).

During the Syrian crisis, as a result of the low level of health awareness, the non-proliferation of programs that devote to mental health, increasing poverty rates, and spreading ignorance, there is a gap in medical literature association between physical activity, sedentary time, and participation in organized activities, social support, sleep problems, and mental distress.

Thus, this study adds to the literature by searching for possible links between these ranges of controllable variables and adding more information about Syria's mental health. Furthermore, due to the special circumstances that Syria is facing from war and economic isolation, this study will allow further understanding of mental distress's causes in the country. Furthermore, it would enable more effective methods to adjust relevant risk factors. As a result, the general Syrian population will achieve better mental health status.

## Methods

### Participants and procedures

This an online survey cross-sectional study aims to gather and assess data on behaviors of health, health in general, wellbeing, and adult population quality of life. Regarding inclusion criteria, a random sample of (1,226) Syrian people (over 18 years old), from all 14 governorates in Syria and were willing to participate were included in the current study. The exclusion criteria were non-Syrian nationality, Syrian nationality who were under the age of 18. They were invited to participate in the current research using social media platforms, including Facebook, telegram, and WhatsApp. Invited participants were given written and spoken details of the study and access to official websites and social media. From March to April 2022, all participants gave permission by completing an online consent form and an online self-report questionnaire. The completing of the online survey took between 4 and 10 min.

Our survey was pretested and edited accordingly before the beginning of data gathering. The survey's language was clear Arabic, which is easily understandable among the Syrian population. The current study was conducted voluntarily, and all participants had the option to withdraw at any moment. Eight unpaid collaborators were in charge of data collection and anonymization. Researchers who were not engaged in the data collection process or had access to personally identifiable information analyzed the data. Researchers from Aleppo university held legal responsibility for the public health survey. A supervising investigator who functioned as the data collection's mentor was given access to a Google form to guarantee that no fabricated data were included. Both the University of Aleppo and the University of Damascus' ethics committees granted permission for the study and approved it. The research was done in accordance with the Helsinki Declaration.

We estimated the sample size using Calculator.net, which is available at “https://www.calculator.net/sample-size-calculator.html.” The Syrian population was estimated to be 18 million in 2019 by the United Nations. Statistical power analysis was used for sample size calculation with a population proportion of 50%, a 0.05 margin of error, and a 95% confidence level, and the recommended size of the sample was 385.

### Measures

This table contains the questionnaire of our study, answer options, and variables' definitions.

The Hopkins Symptom Checklist (HSCL-5) was used to identify participants with mental distress. The Hopkins Symptom Checklist (HSCL-5) has been proved to be a viable and reliable instrument for evaluating anxiety and depressive symptoms, having been updated from the original 25-item form (18, 19). Every one of the five objects used to assess mental discomfort was given one of the following codes: “not bothered” (coded 1), “a little bothered” (coded 2), “somewhat bothered” (coded as 3), or “Extremely bothered” (4). The overall HSCL-5 score was calculated by summing the scores for each topic and dividing it by the number of questions. Respondents who did not reply to all HSCL questions were excluded from the analysis. A validated cut-off score of (> 2.0) was used to identify patients experiencing mental discomfort after the variable was dichotomized (19).Three questions indicating frequency, intensity, and duration were used to measure physical activity levels during Leisure-time. The data were analyzed in the following way: How often do you typically exercise (Average)? was used to measure physical activity frequency. The different responses were coded: “Never” (coded 0), “less than once a week” (0.5), “once a week,” “2–3 times a week”(20), (2.5), “4–5 times a week” (4.5), and “approximately every day” (6.5). The question “How long do you generally exercise each time?” was used to determine duration (Average). The response alternatives were coded: “Less than 15 min” (coded: 8), “15–29 min”(21), “30 min-1 h” (22), and “more than 1 h (75).” The severity was measured by asking, “How hard do you usually exercise?” (Average). The following were the response options: “Easy without being short of breath or sweaty,” “being short of breath and sweaty,” or “almost completely exhausted.” In addition, data on the frequency, duration, and intensity of leisure-time physical activity were combined to identify people who met the World Health Organization's weekly guidelines of 150 min of moderate-intensity aerobic physical activity or 75 minutes of vigorous-intensity aerobic physical activity (23). A single question indicating hours of sedentary time per day was used to analyze information regarding sedentary time. A trim of 8 hours was chosen based on the results of another research (24), which revealed that those who sat for < 8 h per day were less sad and anxious and had higher levels of vitality than those who sat for more than 8 h per day.

This research assessed involvement in organized activities using two questions representing the frequency of engagement in different activities. The answer options for both items were coded as follows: “never” or “rarely” (coded: 0), “1–3 times per month” (18), “weekly” or “daily” (25). The overall score for involvement in organized activities was computed by adding both items, giving a score between 0 and 4. Also, the variable was subcategorized, and < two points (i.e., < two times/month) were used to identify people who had poor engagement (irregular participation) in organized activities.

The Oslo Social Support Scale (OSSS-3) is a straightforward and cost-effective measure for measuring social support. It comprises three main components: (1) the number of close confidants, (2) the sense of caring for others and connecting with neighbors, and (3) the availability of practical assistance. The following were the coded response options for the first item: “No one” (coded: 1), “1–2” (25), “ “3–5(26), and” 6 or more” (22), The second item's answer possibilities were coded: “no interest” (coded 1), “little interest” (25), “neither great nor little interest” (26), “some interest” (22), “great interest” (27). The third item's answer possibilities were coded as follows: “very difficult” (coded:1), “difficult” (25), “neither easy nor difficult” (26), “easy” (22), and “very easy” (27). By adding the scores from each component, the OSS-3's overall score was computed. According to prior studies (23), in the current study, a score of 12 was used to identify participants who did not have high perceived social support, referred to as low perceived social support.

A single question concentrating on individual experience with sleep disturbances in the preceding week was used to assess sleep disturbances. The four response options ranged from “not at all disturbed” to “very upset.” In this study, participants who said they were “pretty much troubled” or “very much bothered” were determined to have sleep issues.

We used Central Population Register to gain insights into age and gender. We used age as a continuous variable. We used Information about the perceived financial situation as an indicator of socioeconomic status. Perceived financial difficulties were equivalent to “very difficult” and “difficult” responses.

### Pilot study

We administered this questionnaire to 50 randomly selected members of the general public to demonstrate its suitability and readability. Then, we made modifications based on participant feedbacks. Then, employing 50 participants, we conducted a pilot test to assess the questionnaire's reliability. Using the Cronbach's alpha test, the internal consistency of each set of questions for the evaluation behaviors of health, health in general, wellbeing, and adult population quality of life was adequate. The scale of HSCL-5 had a reliability coefficient of 0.746.

### Statistical analyses

The age variations between men and women were examined using an independent sample *t*-test. In contrast, financial position, mental distress, leisure-time physical activity, sedentary time, involvement in organized activities, and sleep issues were all evaluated using Pearson chi-square tests (Table 1).We checked the normality of the data, that was non-parametric after performance of Shapiro–Wilk test Pearson chi-square tests were also used to identify men and women with low levels of leisure-time physical activity, high levels of sedentary time, low levels of involvement in organized activities, low levels of social support, and sleep difficulties (Table 2).

**Table 1:** Differences in mental distress between men and women with low level of leisure-time physical activity and high sedentary time.

	**Low leisure-time physical activity % (95% CI)**			**High sedentary time % (95% CI)**		
	**Men**	**Women**	*P*-values^*^	**Men**	**Women**	*P*-values^*^
Mental distress	11 (9.25–12.75)	38.3 (35.28–40.72)	0.543	11.8 (10.18–13.82)	32.2 (23.39–34.61)	0.012
No mental distress	12.8 (11.12–14.88)	38 (35.28–40.72)		20.6 (18.72–23.28)	35.4 (32.33–37.67)	

^*^ χ^2^tests were used to analyze differences in mental stress according to gender.

**Table 2:** Differences in mental distress between men and women with low participation in organized activities, low social support and sleep problems.

	**Low participation in organized activities % (95% CI)**			**Low social support % (95% CI)**			**Sleep problems % (95% CI)**		
	**Men**	**Women**	*P*-values^*^	**Men**	**Women**	*P*-values^*^	**Men**	**Women**	*P*-values^*^
Mental distress	9.8 (7.40-10.60)	28.8 (26.46-31.54)	0.029	10.1 (8.32-11.68)	28.7 (25.48-30.52)	0.023	11.7 (9.25-12.75)	41 (38.24-43.76)	0.190
No mental distress	19.3 (16.80-21.20)	42.1 (39.23-44.77)		19.8 (16.80-21.20)	41.5 (38.24-43.76)		13.1 (11.12-14.88)	34.3 (31.35-36.65)	

^*^χ^2^tests were used to analyze differences in mental stress according to gender.

Furthermore, we investigated potential associations between low leisure-time physical exercise, high sedentary time, low engagement in organized activities, inadequate social support, sleep disturbances (independent variables), and mental distress (dependent variable) using crude multivariable logistic regression models and models adjusted for age and perceived financial situation (Table 3). Models for men and women were shown separately. Pearson correlation tests found that the independent variables in the models had low pairwise correlations, indicating that multicollinearity was not present. The level of statistical significance was chosen at *p* = 0.05 for data analysis using IBM SPSS version 25.

**Table 3:** Adjusted odds ratio (OR) and 95% confidence interval (CI) for mental distress in relation to low level of leisure-time physical activity, high sedentary time, low participation in organized activities, low social support and sleep problems among adults.

	**Model 1**	**Model 2**	**Model 3**	**Model 4**	
	**Men OR (95% CI)**	**Men** **OR (95% CI)**	**Women OR (95% CI)**	**Women** **OR (95% CI)**	
**Exposure variables**	**Leisure-time physical activity (PA)**				
	High PA	Ref.	Ref.	Ref.	Ref.
	Low PA	2.00 (1.18-3.37)^*^	1.96 (1.13-3.40)*	0.54 (0.39-0.72)^***^	0.54 (0.39-0.74)^***^
	**Sedentary time**				
	Low sedentary time	Ref.	Ref.	Ref.	Ref.
	High sedentary time	1.09 (0.70-1.72)	1.11 (0.69-1.78)	1.69 (1.26-2.25)^***^	1.79 (1.32-2.41)^***^
	**Organized activities**				
	High involvement	Ref.	Ref.	Ref.	Ref.
	Low involvement	1.03 (0.47-2.25)	0.91 (0.41-2.06)	1.15 (0.53-2.47)	1.25 (0.57-2.76)
	**Social support**				
	Strong social support	Ref.	Ref.	Ref.	Ref.
	Low social support	0.81 (0.30-2.15)	0.90 (0.31-2.53)	0.73 (0.31-1.75)	0.83 (0.33-2.09)
	**Sleep problems**				
	No sleep problem	Ref.	Ref.	Ref.	Ref.
	Sleep problems	2.34 (1.45-3.77)^***^	2.19 (1.32-3.63)**	2.71 (2.02-3.64)^***^	2.83 (2.09-3.85)^***^
**Control variables**	**Age** ^**a**^		0.98 (0.94-1.03)		1.03 (1.01-1.07)^*^
	**Residence**				
	Countryside		Ref.		Ref.
	City		0.81 (0.46-1.42)		1.24 (0.87-1.76)
	**Displaced**				
	No		Ref.		Ref.
	Yes		0.79 (0.41-1.53)		0.83 (0.55-1.26)
	**Relationship status**				
	Married		Ref.		Ref.
	Single		0.68 (0.28-1.63)		0.52 (0.29-0.93)
	**Worked in health sector**				
	No		Ref.		Ref.
	Yes		0.70 (0.42-1.18)		0.74 (0.51-1.09)
	**Perceived work nature**				
	Easy		Ref.		Ref.
	Difficult		1.73 (0.38-7.80)		6.87 (2.45-19.23)^***^
	**Had Chronic disease**				
	No		Ref.		Ref.
	Yes		0.97 (0.45-2.11)		0.79 (0.47-1.35)
	**BMI** ^**b**^		1.01 (0.98-1.02)		0.99 (0.98-1.01)
	**Perceived financial condition**				
	Easy		Ref.		Ref.
	Difficult		0.55 (0.30-1.03)		0.66 (0.47-0.92)

^*^p < 0.05, ^**^p < 0.01, ^***^p < 0.001. ^a^Age was modeled as a continuous variable and that the odds ratio is for a 1-year increment in age. ^b^BMI was modeled as a continuous variable and that the odds ratio is for a 1-unit increment in BMI.

## Results

### Response rate

A total of 1,224 responses were collected, with 371 men and 853 women participating. All participants fully completed the questionnaire, resulting in a 99 % response rate.

### Demographics

Age, gender, relationship status, chronic disease, residency status, and BMI of the participants are listed in Table 4, which revealed higher levels of mental distress in females than males; in addition, compared to men, women report lower levels of physical activity during leisure time, lower engagement in organized activities, sleep disturbances and challenging job environments. At the same time, no gender differences were detected in high sedentary time or low social support.

**Table 4:** Descriptive statistics of respondents (*N* = 1,224).

	**Sample characteristics**		
**Variables**	**Men (*N* = 371)**	**Women (*N* = 853)**	***P*-values**
Age, mean (SD)	24.81 (7.31)	24.04 (6.79)	0.057
Being city residence, *n* (%)	278 (22.7)	653 (53.3)	0.541
Being displaced, *n* (%)	54 (4.4)	130 (10.6)	0.758
Being single, *n* (%)	273 (23.7)	691 (60.0)	0.175
Worked in health sector, *n* (%)	134 (10.9)	321 (26.2)	0.615
Difficult work nature, *n* (%)	70 (5.7)	90 (7.4)	0.000
Had chronic disease, *n* (%)	39 (3.2)	72 (5.9)	0.246
BMI, mean (SD)	25.21 (10.54)	23.18 (10.49)	0.002
Mental distress, *n* (%)	126 (10.3)	346 (28.3)	0.029
Low leisure-time physical activity, *n* (%)	81 (6.6)	263 (21.5)	0.001
High sedentary time, *n* (%)	178 (14.5)	374 (30.6)	0.182
Low participation in organized activities, *n* (%)	338 (27.6)	823 (67.2)	0.000
Low social support, *n* (%)	352 (28.8)	827 (67.6)	0.076
Sleep problems, *n* (%)	106 (8.7)	323 (26.4)	0.002
Hard financial condition, *n* (%)	76 (6.2)	247 (20.2)	0.002

### Mental distress between men and women with a low level of leisure-time physical activity and high sedentary time

Following the results in Table 4, Low levels of physical exercise during leisure time and prolonged periods of sedentary were also identified as possible risk factors for mental health. According to Table 1, women with mental distress have a greater level of high sedentary time than men (32.2 vs. 11.8 respectively), with (*P*-values of 0.012). Thus, high sedentary time was related to gender when dealing with mental distress. However, there was no significant gender difference between mental distress and low leisure-time physical activity, and both were independent as no link was established (*P*-values 0.543).

## Discussion

This cross-sectional study found a significant correlation between behavioral risk factors and mental discomfort in a randomly selected sample of Syrian individuals above 18 years. After adjusting for potential variables, our findings demonstrated a relationship between limited free-time workouts and mental anguish. According to a prior meta-analysis, working out appears to lower depression with an intermediate impact and anxiety with a modest effect in non-clinical populations (28). According to a meta-analysis study, which supports our findings, exercise may have a comparable potential preventative effect on depression symptoms in both men and women (29, 30). Working out appears to have a mutual relationship with depression; it may prevent and relieve depressive manifestations in the general community, while having depressive manifestations in young people may be seen as an obstacle to physical activity (31). Previous research indicates that regular workouts stimulate a mechanism that enhances emotional pliability to stress (32, 33). However, it is probable that the factors underlying the observed effects of physical exercise on mental suffering are complex, including both psychological (e.g., boosting enthusiasm, emotions of proficiency, and self-effectiveness) and neurophysiological (e.g., increasing production and secretion of neurotransmitters and neurotrophic factors linked with neurogenesis, neovascularization, and neuroplasticity) (24, 34–39). Furthermore, our study found a link between excessive inactivity levels (8 h per day) and mental distress in both males and females. Systematic reviews have found that inert time has a slight positive relationship with anxiety and depression manifestations. Still, they have also found that a few research papers have looked into this relationship, with somewhat conflicting results (40, 41). Additionally, prior cross-sectional surveys have demonstrated a correlation between excessive inactive time and mental anguish (22, 42). One potential reason for the observed relationship between inactive time and mental anguish is that inactivity may replace exercise, which has been shown to reduce levels of mental illnesses (43). A physically inactive lifestyle may be a key risk element for mental illness, as continuous inactive time appears to decrease and impair insulin tolerance modifications, glucose homeostasis, and plasma triglyceride levels, which could consequently affect one's frame of mind and happiness (44). According to studies, engaging in structured events gives people a feeling of closeness, worth, and bonding (45). and may favor mental stability. Whereas, some research has concentrated exclusively on whether involvement in physical exercise groups may be related to enhanced mental balance, our study used a spacious method to develop cooperation in structured events by focusing on participation in workout groups, civic teams, tender work, spiritual societies, and associations. According to the findings of this study, limited participation in structured events was correlated with an elevated risk of mental anguish in both males and females. These results are testified by findings from prospective studies among European adults, which show that participation in socially structured events predicts wellbeing and a reduction in the level of symptoms of depression over time, in addition to serving as a preventative measure against the beginning or progression of chronic diseases, particularly in those with few close social connections (9, 46). Consequently, a Norwegian population-based study found that engagement in organized activities had a concentration-response impact on comprehended health, anxiety, depression, and contentment in both females and males and that males who participated in receptive, rather than innovative, civilizing programs, had favorable wellness status (47). Nevertheless, not many rigorous experimental studies have examined the gender-specific effects of participating in various structured events on mental wellness. According to our findings, various previous researchers have found that minimum limits of sensed social support are related to mental anguish in both males and females (48, 49). Additionally, among elderly people who wanted welfare assistance, females appeared to require more passionate assistance than males, but males appeared to require more noticeable help (48). A linear study of British individuals determined a mutual link between social support and mental wellness, which fluctuated throughout their lives (50). Moreover, insufficient social cohesion between peers in adolescence has lately been bound up with a higher risk of depression in US adults (51), and systematic review studies have shown evidence relating a lack of welfare aid, lack of communication, and aloneness to mental illnesses (52, 53). Another aspect is that traits such as trusting people, a sense of safety in society, and having social communication have been linked to a lower likelihood of mental anguish (54). Previous research has suggested that high levels of observed welfare aid can reduce the possibility of mental disturbance caused by adverse lifetime circumstances, including unemployment and divorce (55, 56). Eventually, the current study found that males and females with sleeping disorders were more prone to intellectual disturbance. Former research found a U-form relationship between sleep time and mental disturbance (57), and people who had both wakefulness and a short sleep time (< 7 h) had a higher risk of persistent mental wellbeing manifestations (58). Furthermore, a study of elderly Norwegian found that various manifestations of wakefulness appeared to have a vital role in the rise of anxiety extents (59), and a meta-analysis found that people who had insomnolence but had no depression had a double risk of emerging depression as compared with those who did not have sleep problems (60). Studies have also demonstrated that sleep disorders and mental anguish have a two-sided causality, with mental anguish raising the possibility of insomnolence in the future (61, 62). The fundamental psychophysiological principles that anticipate mental anguish through insomnolence are still unknown. Previous research has emphasized the function of sleep in feelings control (58–63). A malfunction in sleep-wake controlling brain networks may give rise to changes in feelings sensitiveness from a neurologic standpoint (64). The current study has several methodological flaws that should be evaluated. The cross-sectional design of this study is its fundamental flaw, as it prevents causal inferences. Furthermore, the current study used the abovementioned scales, which are susceptible to memory and remember tendency and socio-occupational tendency. One of the present investigation's main advantages was the big sample randomly selected from a vast community. Another advantage is that we used proven techniques to measure mental distress (HSCL-5) and welfare help (OSS-3) (65, 66). as well as data on frequency, length, intensity, and duration of leisure-time workouts, to identify those who met the WHO recommendations (67) Additionally, because observed economic challenges have been shown as a critical cause of mental anguish (68), they were used to measure socioeconomic situation in this survey.

### Limitations

Our study is a national cross-sectional study, and data collection was done *via* online google forms. Thus, we are unable to prevent some biased or unorganized answers, since the survey was sent through a Google Form to social networking platforms, making it impossible for the elderly, those living in rural areas, and people who are illiterate, do not have access to the internet, or do not have email, to take the survey. In addition, we didn't insert additional information on the context in the country, for example exposure to trauma, and considering that the country has been devastated by conflict and war. We have tried to minimize the biased data by distributing the survey equally between all Syrian counties and taking equal answers from both sexes. As a significant limitation in our research, sleep disruption is an essential variable in mental health and reliable and accurate questionnaires are available in this aspect, so utilizing one question to assess this condition may not be acceptable to identify a link to mental anguish, but we have relied on a scientific reference to assure this association by this methodology. Even though we listed a lot of limitations, there are also a lot of strengths, such as the fact that we distributed the questions around and collect answers from all Syrian governments, as well as high response rate and a large sample size representative of the total Syrian population along with variable data collected from different regions in Syria.

## Conclusions

High sedentary time was found to have measurable links with gender deference as women with high sedentary time had demonstrated more mental distress than men with high sedentary time. Limited involvement in organized activities and low leisure-time physical activity may be risk factors for mental health discomfort in our study's female participants. Additionally, sleep problems and hard financial were observed in subjects with mental illness of both sexes. We advise more studies to be held to investigate why Syrian women's mental health is more vulnerable when dealing with these variables.

## Data availability statement

The raw data supporting the conclusions of this article will be made available by the authors, without undue reservation.

## Ethics statement

The studies involving human participants were reviewed and approved by Aleppo and Damascus Institutional Review Boards. All methods were carried out in accordance with relevant guidelines and regulations or the Declaration of Helsinki. The patients/participants provided their written informed consent to participate in this study.

## Author contributions

SSw: conceptualization, methodology, formal analysis, writing-original draft, review, and editing. HA, HB, AN, ME, MAlm, SK, BS, MAlb, EB, NE, SA, EA, MH, MP, and SSo: writing—review and editing. WH: proofreading, language editing, and conducting the reviewers comments. All authors contributed to the article and approved the submitted version.

## Data collection group

-Hala Almohi Alsaid Mushaweh

halamshaweh@gmail.com

Faculty of Medicine, Aleppo University, Aleppo, Syria

- Odai Maihoub

Odai.maihoub1@gmail.com

Faculty of Medicine, Tishreen University

- Yara Ebrahem

yaraebrahem246@gmail.com

Faculty of Medicine, Damascus University

- Eskandr Rappoh

eskandr8@gmail.com

Faculty of Medicine, Tishreen University

- Enas AlShbib

enas.shbeeb.99@gmail.com

Faculty of Medicine, Aleppo University

- Zeina Yassen

zzeze2079@gmail.com

Faculty of Health Sciences, Al Baath University

- Nour Jreikh

nour2000j1@gmail.com

Faculty of Medicine, Aleppo University

- Mohammad Zakaria Kharboutly

Zkareakharboutly@gmail.com

Faculty of Medicine, Aleppo University
